# Geodetic evidence for interconnectivity between Aira and Kirishima magmatic systems, Japan

**DOI:** 10.1038/s41598-018-28026-4

**Published:** 2018-06-28

**Authors:** E. Brothelande, F. Amelung, Z. Yunjun, S. Wdowinski

**Affiliations:** 1 Department of Marine Geosciences, Rosenstiel School of Marine and Atmospheric Science, University of Miami, 4600 Rickenbacker Causeway, Miami, FL 33149 USA; 2 Department of Earth and Environment, Florida International University, Miami, Florida USA

## Abstract

It is not known whether clustered or aligned volcanic edifices at the Earth surface have connected magmatic systems at depth. Previously suggested by geological records of paired eruptions, volcano interconnectivity still lacks proper geodetic evidence. Here we use GPS time-series and deformation modeling to show how Aira caldera and Kirishima, two adjacent volcanic centers in Kagoshima graben (southern Japan), interacted during Kirishima unrest in 2011. Whereas Aira caldera had been inflating steadily for two decades, it deflated during the eruption of Kirishima which started with a large-volume lava extrusion. This deflation, which cannot be explained by stress changes, is interpreted as the result of magma withdrawal from the Aira system during the Kirishima replenishment phase. This study highlights the behavior of connected neighboring volcanic systems before and after a large eruption, and the importance of taking into account volcano interactions in eruption probability models.

## Introduction

Recent studies have widely improved our understanding of how volcanoes respond to tectonic^1–4^ and climatic^5–7^ forcing. Yet, little is still known about how volcanoes interact and whether neighboring volcanoes represent individual separated plumbing systems or the surface expression of a large connected magmatic system at depth.

The existence of volcano interactions has been strongly suggested by geological studies in a few cases of large eruptions such as paired eruptions at Taupo volcanic zone^8^, concurrent subsidence and resurgence at Uncompahgre and San Juan calderas^9,10^, and the caldera collapse at Mount Katmai in 1912 related to a lateral magma withdrawal feeding the Novarupta eruption^11^. Several attempts of evidencing interactions from geodetic signals have been made. A temporal correlation was established between the onset of an eruption at Kilauea and the beginning of an inflation phase at Mauna Loa in 2002, which was interpreted to be caused by a common magmatic pulse^12^. However, other authors argued that the inflation phase at Kilauea actually started 6 months earlier, so a more complex mechanism was proposed to explain the interaction if there was^13^. A possible coupling between Vesuvius and Campi Flegrei was proposed based on concomitant uplifts at both edifices shown by InSAR^14^. However, ambiguity remains because of signal uncertainties on Vesuvius and possible atmospheric effects, later recognized to be significant at these volcanoes^15^.

**Figure 1:**
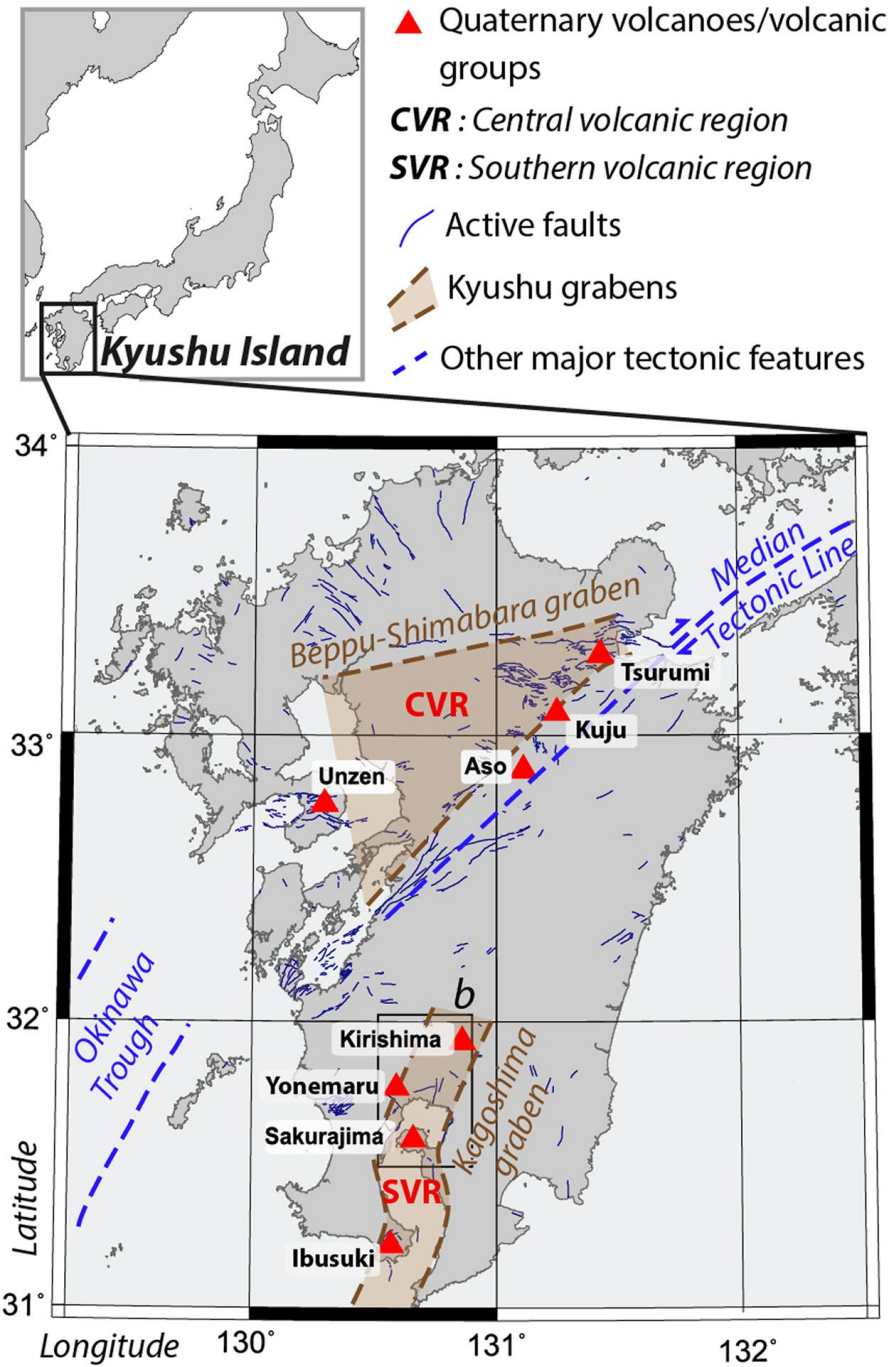
Volcano-tectonic map of Kyushu Island showing Quaternary volcanoes (red triangles) organized in two groups (CVR and SVR) linked to the presence of active grabens. *Inset*: Location of Kyushu Island in Japan.

In Kyushu (Southern Japan), active volcanism results from the subduction of the Philippine Sea plate beneath the Eurasian plate and appears to be fundamentally linked to back-arc extension^16^. Aira caldera and Kirishima volcanic group are located ~22 km away from each other, in the same active graben (Kagoshima, Fig. 1). In this paper, we show how the caldera dynamics was affected by the 2011 eruption of its neighbor, using deformation data inferred from permanent GNSS stations in the area (Supplementary Fig. A.1).

**Figure 2:**
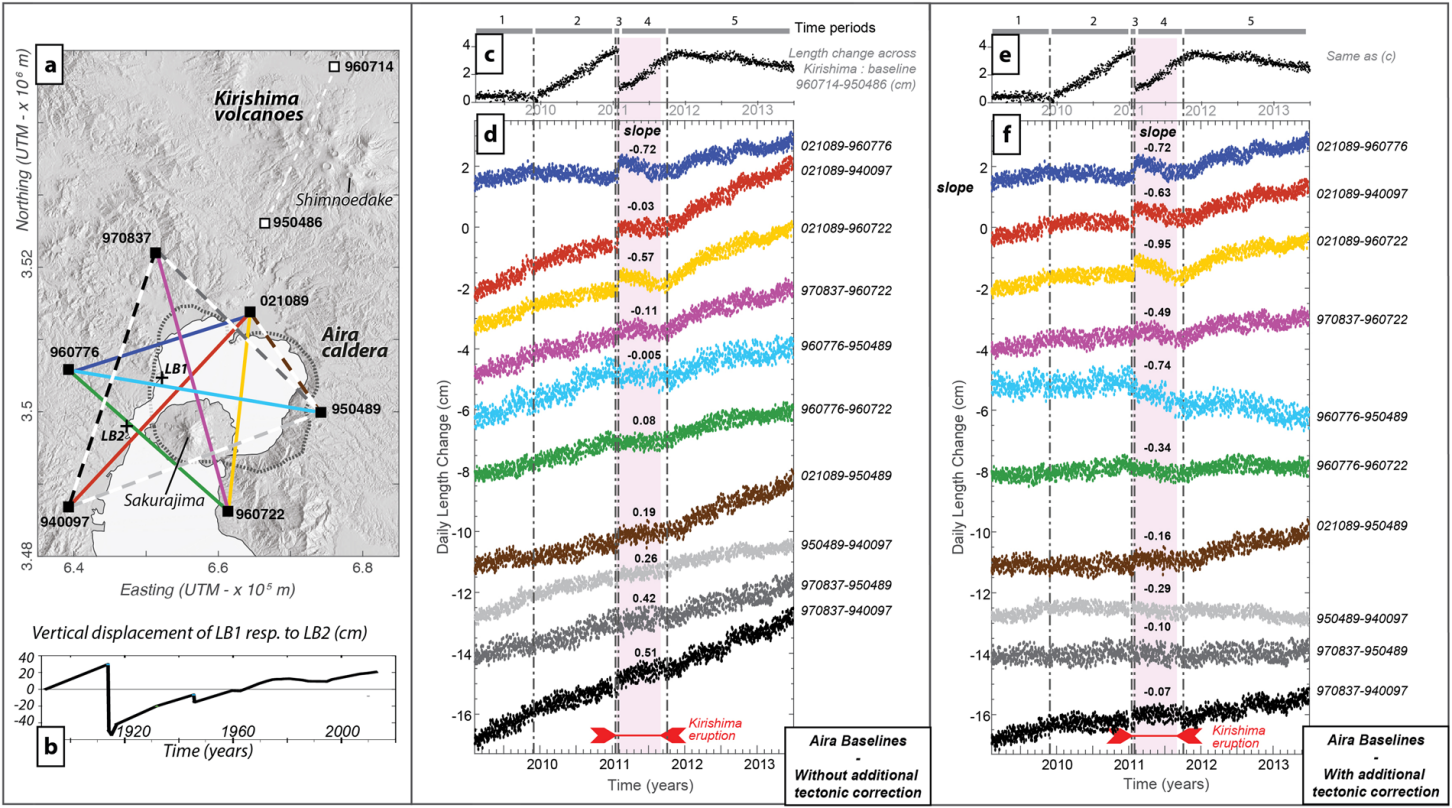
(**a**) Map of Aira caldera and Kirishima volcanic group in Kyushu showing leveling benchmarks (LB) and GPS baselines used in (**b**,**c**,**d**,**e**,**f**) plotted over the shaded relief map of a 10-m resolution Digital Elevation Model (DEM; Source: Geospatial Information Authority of Japan website, see Data Availability). (**b**) Vertical displacement LB1 compared to LB2 showing the caldera floor uplift over the last century^19^. (**c** and **e**) Baseline change across Kirishima (see stations in **a**). (**d**) GPS baseline length evolution over Aira caldera in the case of non-graben corrected data. Color-coded solid and dashed lines on (**a**) correspond to the top 6 and bottom 4 series on (**d**), respectively. Time-periods 1–5 on (**c**) are defined by changes in Kirishima deformation patterns (grey vertical dash-dotted lines). The pink box indicates Shinmoedake eruption. The slopes of linear fits of baseline length changes during period 4 are indicated (cm/year). (**f**) GPS baseline length evolution over Aira caldera in the case of graben-corrected data. Same color-coding and slope indications as in (**d**).

Aira caldera, one of the most active and hazardous volcanoes in South Japan, is associated with an active cone, Sakurajima (Fig. 2a). The activity of Sakurajima, characterized by frequent (daily) vulcanian explosions since 1955, has proven to be strongly related to the caldera dynamics. A Plinian eruption (VEI 4) occurred in 1914, which caused the death of 58 people, after a period of inflation of Aira caldera and was accompanied by ~1-m subsidence of the caldera floor^17–19^ (Fig. 2b). Since then, the caldera has been generally inflating, except for subsidence associated with the 1946 Sakurajima eruption. The caldera floor has now reached approximately the pre-1914 level, raising the threat of a new strong explosive event^20^.

Kirishima is a group of volcanoes, at the north of Aira, among which Shinmoedake produced two strong magmatic or magmato-phreatic eruptions separated by almost 300 years, in 1716–1717 and in 2011^21^. After a small phreatic activity (starting in August 2008) and a pre-eruptive inflation (starting in December 2009), the 2011 eruption started with a series of phreatomagmatic, sub-Plinian and lava dome emplacement events from January 19^th^ to 31^st^. This first phase, called the eruption climax, extruded most of the total erupted volume (21–27 10^6^ m^3^ DRE^22^) and was accompanied by a strong co-eruptive deflation^23^. After the climax, another inflation of Kirishima occurred until November 2011, accompanied by sparser and smaller vulcanian and phreatomagmatic events until September.

## Results

### Baseline changes

We use the length evolution of GPS baselines in Aira caldera area (Fig. 2a), that provide a synthetic view of deformation, to determine whether the magmatic system was affected by the processes associated with the eruption at Kirishima. We consider 5 periods between 2009 and 2013, based on the deformation recorded at Kirishima (a baseline example crossing Kirishima is given on Fig. 2c,e): pre-unrest in which deformation at Kirishima is very limited (period 1), pre-eruptive inflation (period 2), deflation associated with eruption climax (period 3), post-climax inflation (period 4) and post-unrest (period 5). Two sets of baselines are proposed: on Fig. 2d, baselines are corrected for offsets, outliers and seasonal variations, and on Fig. 2f, an additional correction is applied in order to remove a possible contribution of the graben activity to the horizontal displacements of stations 940097, 960722 and 950489 (see Methods).

We consider 6 baselines crossing the central part of Aira caldera and 4 peripheral baselines (solid and dashed lines on Fig. 2a, respectively). The length increase of all baselines over the whole study period on Fig. 2d reflects a general inflation of the Aira caldera area. The first three baselines, linked to station 021089, show a sudden length increase during period 3, clearly associated with Kirishima co-climax deflation. During period 4, all central baselines either decrease or stay constant (the first six on Fig. 2d), which is quantified by the slopes of best linear fits, either negative or very close to 0. Baselines crossing the peripheral part show a very small lull of their length increase rate (last four baselines on Fig. 2d), and slopes remain positive during period 4. These results show a deflation of Aira caldera during period 4 (Kirishima’s post-climax period) with a deflation source located beneath the central part of Aira caldera.

The additional correction used for Fig. 2f reduces the general baseline length increase, notably for E-W oriented baselines such as 960776–950489, 960776–960722, 950489–940097 and 970837–950489, that become mostly flat outside of period 4. This illustrates that the inflation recorded at Aira caldera may be partially of tectonic origin (linked to the graben expansion). Nonetheless, the relative change in Aira dynamics during period 4 remains striking in these time series, with more negative slope values after additional correction. Aira source deflation remains much clearer on central baselines (first six baselines on Fig. 2f, slopes <−0.3 cm/year) than on peripheral ones (last four baselines, slopes >−0.3 cm/year).

### Velocities and estimated volume change rates

**Figure 3:**
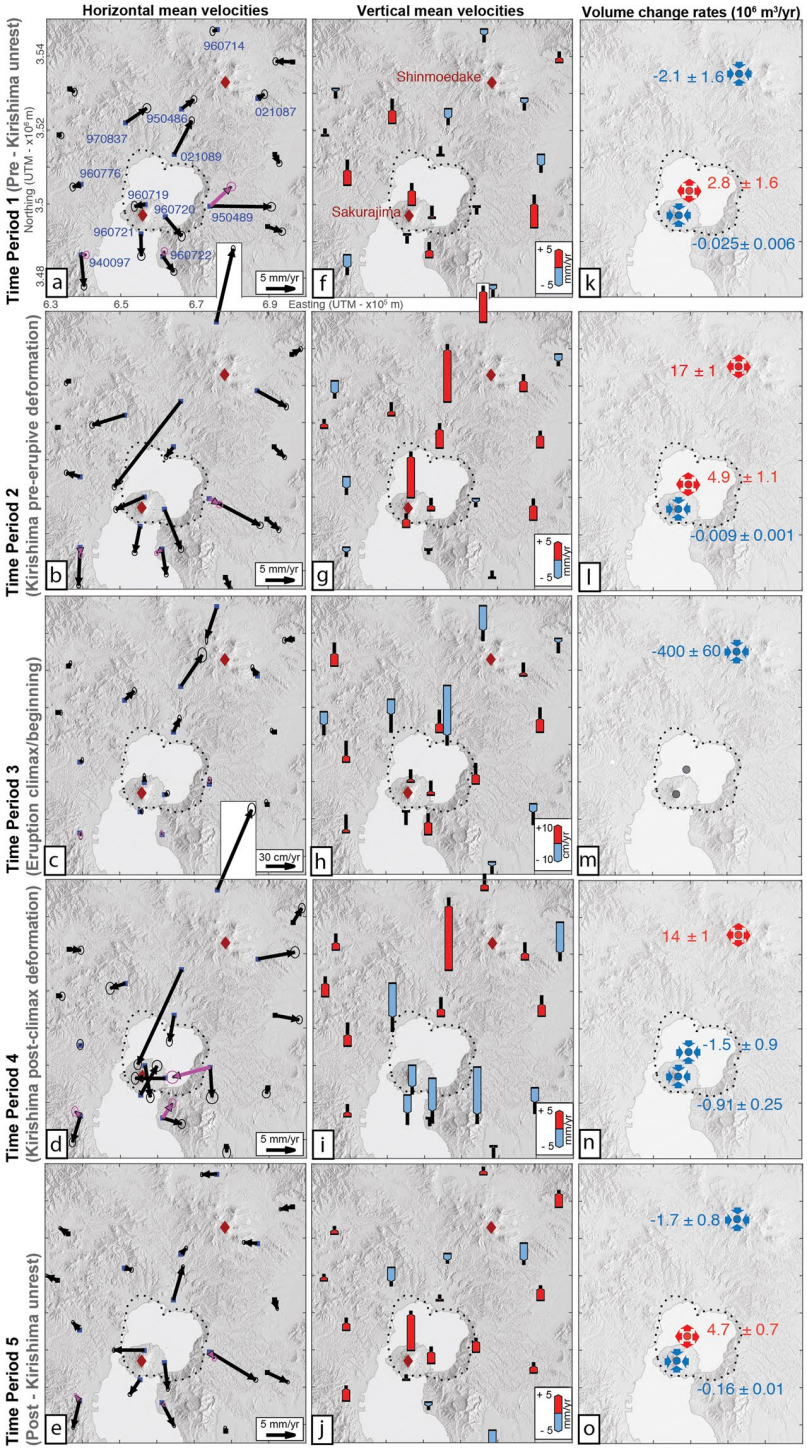
Mean velocities (horizontal: 1^st^ column, vertical: 2^nd^ column) observed at GEONET GPS stations in Aira and Kirishima areas, and best-fitting volume change rates in millions of m^3^/year (3rd column, red for +, blue for −) for time periods 1–5. Pink arrows represent re-estimated horizontal velocities at three stations after the additional tectonic correction (see Methods). Ellipses and black vertical bars represent 1-sigma uncertainties for horizontal and vertical velocities, respectively. Station named on (**a**) are used for triple-source modeling in COMSOL. Red diamonds: Shinmoedake and Sakurajima volcanoes. Black dotted line: Aira caldera rim. Shaded relief maps generated from a 10-m resolution DEM (Source: Geospatial Information Authority of Japan website, see Data Availability).

Horizontal and vertical velocities for the five periods resolve the deformation processes with more detail and give a more complete picture of the deformation pattern on both volcanic systems. GPS velocities were calculated from displacement time series after the removal of offsets, tectonic component, common mode error, outliers and seasonal variations. Horizontal velocities represented by black arrows (Fig. 3a–e) suppose a simple and linear model of tectonic velocities (non-graben corrected data, see Methods). Residual constant velocities are observed at stations 940097, 960722 and 950489; they may be additionally removed in the hypothesis they result from the graben activity: this additional correction is represented by pink arrows for these three stations (graben-corrected data, see Methods). Vertical velocities are represented as blue (when negative) and red (when positive) bars on Fig. 3f–l. Then, horizontal and vertical velocities were used to estimate the volume change rates of 3 magma reservoirs, under Aira, Sakurajima and Kirishima (see Supplementary Table B.1 and Fig. B.1 for source location and comparison between real and model velocities, respectively). We used a finite element model, with sources embedded in an elastic medium in order to take into account source interactions in our inversions (see Methods).

For Kirishima, during periods 2, 3 and 4, the horizontal and vertical velocities show inward, outward, inward (Fig. 3b–d) and upward, downward, upward patterns (Fig. 3g–i), respectively. This indicates a consecutive sequence of source and edifice inflation, deflation and inflation. The deformation center is about 5 km NW of Shinmoedake (in agreement with previous studies^23^). Accordingly, the model shows an expansion of Kirishima source during period 2 (Fig. 3l), a contraction during period 3 (Fig. 3m), and an expansion again during period 4 (Fig. 3n). The inferred volume changes (Table 1) agree with previous estimations obtained from more numerous GPS stations^23^. Periods 1 and 5 lack well defined deformation patterns (Fig. 3a,e,f,j).

**Table 1:** Time periods considered, estimated volume changes (and rates) at the 3 sources inferred from GPS data, and volume changes (and rates) in the Aira-Sakurajima system attributed to Sakurajima ash emissions (see text).

Time period	Dates	Duration in years	Kirishima dynamics	Volume changes in 10^6^ m^3^ inferred from GPS (*and volume change rates in 10*^6^ *m*^3^*/yr*)			Volume changes linked to Sakurajima ash emissions in 10^6^ m^3^ (*and rates in 10*^*6*^ *m*^*3*^*/yr*)
				Aira	Sakurajima	Kirishima	
1	6 Feb 2009–1 Dec 2009	0.82	pre-unrest	2.30 ± 1.3 (*2.82* ± *1.6*)	−0.02 ± 0.01 (*−0.02* ± *0.01*)	−1.75 ± 1.3 (*−2.15* ± *1.6*)	−0.65 (−*0.80*)
2	1 Dec 2009–19 Jan 2011	1.14	pre-eruptive	5.60 ± 1.2 (*4.93* ± *1.1*)	−0.01 ± 0.001 (*−0.01* ± *0.001*)	19.24 ± 1.1 (*16.95* ± *0.9*)	−2.03 (−*1.78*)
3*	19 Jan 2011–31 Jan 2011	0.03	climax	—	—	−13.56 ± 1.9 (*−399* ± 56)	—
4*	31 Jan 2011–4 Oct 2011	0.67	post-climax	−1.00 ± 0.6 (*−1.48* ± *0.9*)	−0.61 ± 0.2 (*−0.91* ± *0.3*)	9.55 ± 0.9 (*14.18* ± *1.3*)	−0.85 (−*1.26*)
5	4 Oct 2011–4 Jul 2013	1.74	post-unrest	8.13 ± 1.3 (*4.67* ± *0.7*)	−0.28 ± 0.02 (*−0.16* ± *0.01*)	−2.93 ± 1.4 (*−1.68* ± *0.8*)	−3.73 (−*2.14*)

No estimation is given for the Aira-Sakurajima system during period 3 due to high uncertainties. (*) Kirishima eruption periods.

The evolution of deformation in Aira caldera and its surroundings is more complex. Horizontal velocities show outward patterns during periods 1, 2 and 5 (Fig. 3a,b,e). Only station 021089 shows an inward velocity during period 2 due to the direct influence of Kirishima pre-eruptive inflation. These horizontal patterns reflect Aira inflation, supported by a consistent uplift of the northern flank of Sakurajima (station 960719) during all these periods (Fig. 3f,g,j), and confirmed by positive estimated volume change rates (Fig. 3k,l,o). Period 2 deserves a little bit of attention. Indeed, baseline evolution during period 2 (Fig. 2d,f) seemed to indicate a weakening of Aira inflation during this period, which was linked to the inward movement of station 021089 and could occur in response to the nearby Kirishima source expansion. But Fig. 3b shows Aira goes on expanding and inflating, with GPS stations showing greater upward and outward velocities during this period 2 in comparison to period 1 (in particular 960719, 960720, 960721). The estimated volume change rate of Aira source is almost twice the rate in period 1 (4.9 10^6^ m^3^/yr compared to 2.8 10^6^ m^3^/yr; Fig. 3k,l, Table 1), which makes it a period of enhanced volume input for both Aira and Kirishima. This shows baselines are not sufficient to give a full picture of deformation dynamics, and the combined action of nearby sources over an area has to be assessed very carefully. Velocities during period 3 are within the noise.

During periods 1, 2 and 5, the Sakurajima source contracted at very low rates. These results are highly dependent on the source depth, which is not well constrained^24^. Though they may differ from other studies that do not take into account source interactions^25^, studies agree on the very small magnitude of volume changes at Sakurajima source in comparison to Aira’s.

During period 4, the three stations on Sakurajima (960719, 960720 and 960721) show downward vertical velocities and horizontal velocities pointing towards the center of the volcano edifice. This pattern indicates a clear contraction of Sakurajima source. Around the caldera, stations 970837 and 950489 show consistent subsidence during this period. Station 021089, with a vertical velocity within the error bar, is influenced by Kirishima deformation. For horizontal velocities, 021089 points to the center of the caldera, as in period 2 but with a greater value, which probably results from the combined action of Kirishima source expansion and Aira source contraction. Stations along the south-eastern caldera rim, 950489 and 960722, do not seems to move inward when the initial correction process (black vectors, non-graben corrected data) is applied. However, we notice the directions of their horizontal velocities during period 4 strongly differ from other time periods. These stations move inward when the alternative secular deformation model is applied (pink vectors, graben-corrected data). During period 4, volume change estimations show both Aira and Sakurajima sources contracted rapidly (total rate of −2.4 10^6^ m^3^/yr; Fig. 3m). Figure B.1 of the supplementary material shows model fitting, constrained by both non-graben corrected and graben-corrected data, results in very small model displacements for stations 950489 and 960722. This may indicate an underestimation of Aira source deflation during period 4.

A comparison between period 2 and 4 shows that Kirishima exhibits similar deformation patterns and volume change rates (16.9 and 14.2 10^6^ m^3^/yr, respectively). Nonetheless, Aira caldera changed from inflation to deflation.

## Discussion

### Hydraulic connection between magmatic systems

**Figure 4:**
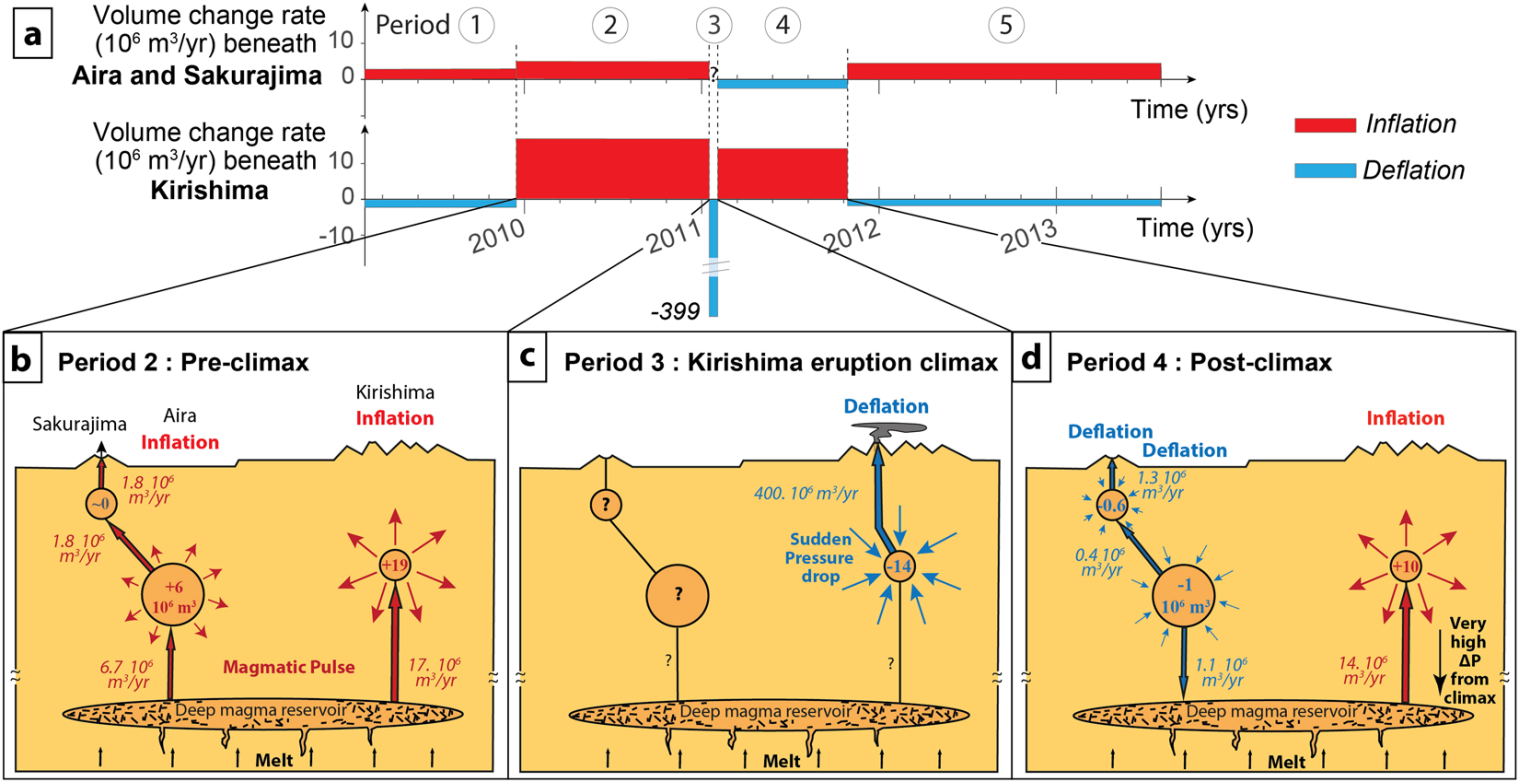
(**a**) Estimated volume change rates (rectangle areas represent total volume changes) during the study period beneath the Aira-Sakurajima system and Kirishima, inferred from GPS. (**b**,**c**,**d**) Schematic sketches illustrating the relative behavior of Aira and Kirishima in the hypothesis of a common deep reservoir before, during and after the eruption climax. Total volume changes are shown inside deformation sources (in units of 10^6^ m^3^). Volume fluxes, inferred from geodetic volume change rates and Sakurajima ash emissions (see Table 1), are shown along conduits (units of 10^6^ m^3^/yr).

The timeline of estimated volume changes for Aira and Kirishima systems indicate a different inflation/deflation history of the sources (Fig. 4a). Throughout most of the 2009–2013 study period, Aira caldera inflated, except for a deflation period in the aftermath of the 2011 eruption of Kirishima volcano. This was Aira’s only deflation during the 2009–2013 study period. In order to better assess the volume budget of the Aira-Sakurajima system, we estimated the volume loss linked to Sakurajima ash emissions at the surface (Table 1) using monthly weights of volcanic ash ejected^26^, assuming a density of 1500 kg/m^3^ and a ratio of erupted volume to geodetic volume change of 2.5 (spherical magma chamber, in which magma compressibility is taken into account^27^). The total volume change of the Aira-Sakurajima system during period 4 (−1.6 m^3^) is twice the volume change attributed to loss through Sakurajima ash emissions (−0.8 m^3^; Table 1). Half of the volume loss thus remains unexplained.

Deflation occurred while the Kirishima magmatic system was recharging following the eruption climax, reflected by rapid ground inflation. Prior to Kirishima’s eruption, both magma systems were inflating simultaneously. The timing of Aira deflation, following the extrusion of a large volume of material at Shinmoedake during the eruption climax, suggests that it is a direct consequence of eruption-related changes to Kirishima’s magmatic system. In the absence of any external event, there are two possible explanations for interaction between neighbouring volcanoes: stress changes and hydraulic connections between the magmatic systems^28^.

Stress change induced by the deformation of Kirishima source can be discarded as an important mechanism in our case. The expansion of Kirishima source should induce a contraction of Aira source. In period 2, we observed the opposite behavior (Aira source is inflating, Fig. 3l) and, in period 4, the estimated contraction is much larger than the expected effect. Indeed, considering a radius ranging from 1 to 5 km for Aira source, the stress-induced volume change at Aira ranges between −2 10^2^ and −2 10^4^ m^3^ (Supplementary Fig. B.2), which is at least two orders of magnitude lower than the estimated volume change of Aira during this time period (−1 10^6^ m^3^, Table 1). We therefore infer that the interaction mechanism involves a hydraulic connection between Aira and Kirishima plumbing systems.

### A common deep reservoir

How can a magmatic connection be maintained over several months and a distance of ~20 km? A crustal sill directly connecting the shallow reservoirs is unlikely because it would freeze rapidly. Another possibility is a deep magma storage extending horizontally over several tens of kilometers feeding the two plumbing systems. Extensive settings (local decompression and high-heat flux) or high-magma productivity zones such as hotspots can explain the presence of such a magma storage. Aira caldera and Kirishima volcanoes are located in the same active graben^16^ (Fig. 1), characterized by a very high heat flux^29^. A continuous electrically-conductive body was imaged by magnetotellurics between Aira and Kirishima, mostly around ~50 km-depth^30^. This could reflect the presence of magmatic fluids connecting the two systems. This depth is also consistent with the roof of a large P-wave attenuation zone beneath Kirishima and Aira^31^. As the depth of the Moho in that region is around 34 km^32^, this deep reservoir would be located in the mantle. At these depths, small volume/pressure changes inside the deep reservoir cannot be detected (and therefore constrained) by ground deformation.

Figure 4(b,c,d) illustrates how the behaviour of Aira and Kirishima sources during the post-climax period can be explained in the hypothesis of a common deep reservoir (fluxes between reservoirs integrate volume change rates inferred from geodetic data and Sakurajima ash emissions). A magmatic pulse from the deep reservoir to shallower levels would lead to volume increases in both sources during period 2 (Fig. 4b), as observed in the data. The higher volume increase rate of Kirishima source in comparison to Aira’s (and higher flux towards Kirishima) may be explained by a wider conduit or a higher pressure-gradient after opening - or reactivation - of a magma pathway.

During the eruption climax (period 3) the large volume erupted by Shinmoedake (21–27 10^6^ m^3^) caused a pressure drop in the Kirishima reservoir (Fig. 4c, volume change of −13.5 10^6^ m^3^; Table 1). The resulting large pressure gradient towards Kirishima can explain the redirection of the flux towards Kirishima reservoir after the climax (period 4, Fig. 4d), depriving Aira reservoir of magma input and even creating a downward magma withdrawal during the time necessary to replenish the Kirishima system. This explains how Kirishima and Aira systems can transition from similar dynamics (both inflating) to opposite ones (one inflating, one deflating). Once Kirishima was replenished, magma input resumed at Aira reservoir (period 5).

In Hawaii, the 6-months delay separating inflation episodes at Kilauea and Mauna Loa, as well as geochemical discrepancies of the lavas, can be explained by a very low hydraulic conductivity of the melt layer connecting the two systems^13^. In our case, no time delay is observed, as Aira deflation takes place right after the lava extrusion phase (climax) at Kirishima and stops almost concomitantly with Kirishima’s recharge. Geochemical studies also show similarities in Sr and Nd isotope ratios for historical lavas at Kirishima and Sakurajima^33,34^, supporting a relatively homogeneous reservoir with high hydraulic conductivity.

## Concluding remarks

Identifying volcano interaction mechanisms is critical to determine if and how an eruption can influence the activity within a given area. We have shown that large volcanic systems such as Aira caldera can respond to small eruptions (VEI 3) at nearby volcanoes fed from a common deep reservoir. However, the volcanic systems are not connected all the time because magma pathways open and close. In the case of Shinmoedake, the vertical connection was closed for around 300 years until it was reactivated. The coupled behavior of Aira and Kirishima most likely represents the clearest example of volcano interconnectivity revealed by geodetic monitoring. The pre-eruptive common magmatic pulse shows that inflation of one volcano can enhance the eruption probability of a neighboring volcano (with or without a delay, depending on the type of connection) while an eruption can decrease that same probability for several months, by redirecting the flux.

## Methods

### GPS data

In order to characterize the deformation associated to Aira caldera and Shinmoedake, we used the data from 32 GNSS stations (12 over volcanic areas and 20 around; Supplementary Fig. A.1) of the GEONET permanent network in southern Kyushu (spatial interval ~20 km^35^). Both baselines lengths and station displacements were used in this study. We used GPS daily position time series provided by the Nevada Geodetic Laboratory^36^ (University of Nevada, Reno - UNR) starting February 2009, approximately 2 years before Kirishima eruption. GPS processing uses the GPS Inferred Positioning System (GIPSY) OASIS II software and final fiducial-free GPS orbit products made available by Jet Propulsion Laboratory. The precise point positioning method is applied to ionospheric-free carrier phase and pseudorange data, and all daily 24 h solutions are aligned to the International GNSS Service 2008 (IGS08) global reference frame (details available at http://geodesy.unr.edu/gps/ngl.acn.txt).

A multistep post-processing was applied in order to isolate the volcanic contribution in GPS position changes. (1) Offsets (earthquake-related, material-related or of unknown cause) were first removed from time series of all three components (eastern, northern and vertical). Their detection relied on manual inspection, which remains the most reliable method^37^ when the amount of data allows it. (2) Secular displacements were estimated and removed, using a model calculated from all stations in Kyushu located south of 32° latitude, except the stations within a distance of 15 km from the two volcanic centers. The 32°latitude limit had to be put because secular displacements above this line show a complex anticlockwise rotation^38,39^. The same methodology was followed to remove remaining transient displacements (ramp on the northern component) related to the Tohoku earthquake (March 11th, 2011). (3) The stations outside the volcanic area were also used to evaluate the common mode errors^40^, i.e. variations that can be observed at the same time at all GPS stations on a regional scale (including all kinds of non-corrected errors). (4) Time series were then cleaned from remaining data outliers. We defined two kinds of outlier points: first, measurements associated with a standard deviation provided by UNR greater than 3 times the mean standard deviation in the individual time series, and second, points that individually deviate from the mean of the time-series (defined by a 20-days sliding window) by more than 2 times the RMS scatter. (5) Finally, annual and semi-annual periodic fits of time series (outside of the deformation and eruption time period) were used to estimate locally and remove seasonal variations at every station.

A complexity appeared for 3 stations along the south and south-eastern rim of the caldera (940097, 960722 and 950489). After the 5-step correction process, their residual horizontal velocities showed a constant component. This constant component may be due to a constant action of a magmatic source or, given the location of these stations, it may indicate an uncorrected secular or long-term velocity related to the activity of the Kagoshima graben. Consequently, we considered an alternative correction in which the secular deformation model includes these residual velocities. As it is not possible to determine whether these residual velocities are of volcanic or tectonic origin, both correction possibilities (representing two end-members) are proposed for baseline length and velocity calculation. We refer to the data obtained after the correction steps described in the previous paragraph as “non-graben corrected”, and to the data after the correction steps along with the removal of constant velocity component at stations 940097, 960722 and 950489 as “graben-corrected”. Both graben-corrected and non-graben corrected data were given a similar weight (50%) to constrain the model inversions.

Baselines length changes provide a synthetic vision of temporal changes over a certain area, and were used to define time periods according to the deformation dynamics of Kirishima and Aira. In order to observe only volcanic contributions, baseline length time series were obtained after applying correction steps 1, 4 and 5 (removal of non-seismic offsets, outliers and seasonal variations; see Fig. 2d). Indeed, we considered that baseline calculation automatically corrects - or reduces to a negligible level – the contribution of tectonic displacements (secular and transient) and common mode errors. However, in the hypothesis that residual velocities observed at stations 940097, 960722 and 950489 are related to the Kagoshima graben activity, we proposed a second set of baseline length changes in Fig. 2f in which the graben correction is applied (residual velocities for the three stations are removed before baseline calculation).

Baselines are less precise than absolute displacements as they integrate three components and they are insensitive to movements in perpendicular directions (in particular, they are relatively insensitive to vertical movements). Therefore, we used absolute horizontal and vertical velocities to provide a complete description of the deformation patterns and to constrain the associated source modeling. Horizontal velocities corresponding to non-graben corrected data are shown as black arrows on Fig. 3a–e. Horizontal velocities corresponding to graben-corrected data are shown as pink arrows for stations 940097, 960722 and 950489 on the same figure.

### Modeling approach

We explain the observed deformation for each time period as caused by volume changes of magma reservoirs. We consider three sources with fixed locations embedded in a homogeneous, isotropic elastic medium (Young’s modulus E = 30 GPa, Poisson’s ratio ν = 0.25). For Kirishima and Aira, we use best-fitting source locations, stable over time, published in the literature^23,24^ and for Sakurajima we use a depth of 4 km, the midpoint of published estimates^24,41^ (Supplementary Table B.1). These locations are supported, in the case of Aira and Sakurajima, by seismic attenuation studies^42,43^.

To account for source interaction effects^44–46^, we use a finite element method as implemented by the COMSOL® multiphysics software. We consider large box dimensions (*90 km* × *110* *km* × *40* *km*) with fixed surface at the bottom, free on the top and roller on the side. Source sizes have minor impact on estimating volume changes, but they are important for static stress transfer effects, so different radii from 1 to 5 km were considered for Aira caldera source.

We consider velocities only at stations close to the volcanic centers (indicated by names on Fig. 3a), invert for the volume change rates using derivative-free methods (BOBYQA and Coordinate search in COMSOL®), and estimate the uncertainties from the linearly propagated uncertainties of the velocities (corresponding to 1 standard deviation).

Our numerical models use simple assumptions, spherical sources in an elastic medium, for two reasons. First, other source shapes were tested (e.g. oblate reservoir beneath Aira^20^) but they did not fit our data as well as spherical sources. Second, more complex assumptions would certainly change absolute values of volume changes, but they would not affect much the relative variations between time periods which is the focus of this study.

### Data availability

The GPS data used in this study is publicly available on the University of Nevada website (http://geodesy.unr.edu). The Matlab R2016a^©^ code and associated functions used for post-processing and plotting are available upon request to the authors. The topographic information used for generating shaded relief maps on Figs 2a, 3, A1 and B1 (Supplementary material) is a 10-m resolution DEM provided by Geospatial Information Authority of Japan (in Japanese; https://na01.safelinks.protection.outlook.com/?url=https%3A%2F%2Ffgd.gsi.go.jp%2Fdownload%2Fmenu.php&data=02%7C01%7Cebrothelande%40rsmas.miami.edu%7C2b7dc5ed125b4bed0bd108d5a5baa06f%7C2a144b72f23942d48c0e6f0f17c48e33%7C0%7C0%7C636597143364499667&sdata=f%2FDi1DA1G42vTxlTc2vgXYFMbAM5x%2B7POynk7mr1PEM%3D&reserved=0).

## Electronic supplementary material

Supplementary material
