# Supplementary material for: Geodetic evidence for interconnectivity between Aira and Kirishima magmatic systems, Japan
Source: Sci Rep. 2018 Jun 28;8:9811. doi: 10.1038/s41598-018-28026-4 (PMC6023929; doi:10.1038/s41598-018-28026-4)
Supplement: Supplementary file 1 — Supplementary material [file 41598_2018_28026_MOESM1_ESM.pdf]

Supplementary material for:

Geodetic evidence for interconnectivity between Aira and Kirishima magmatic systems, Japan

Brothelande, E.[1]*, Amelung, F. [1], Yunjun, Z. [1], Wdowinski, S.[2]

(1) Department of Marine Geosciences, Rosenstiel School of Marine and Atmospheric Science, University of Miami, 4600 Rickenbacker Causeway, Miami, FL 33149, USA
(2) Department of Earth and Environment, Florida International University, Miami, Florida, USA

Appendix A of the supplementary material: GPS data

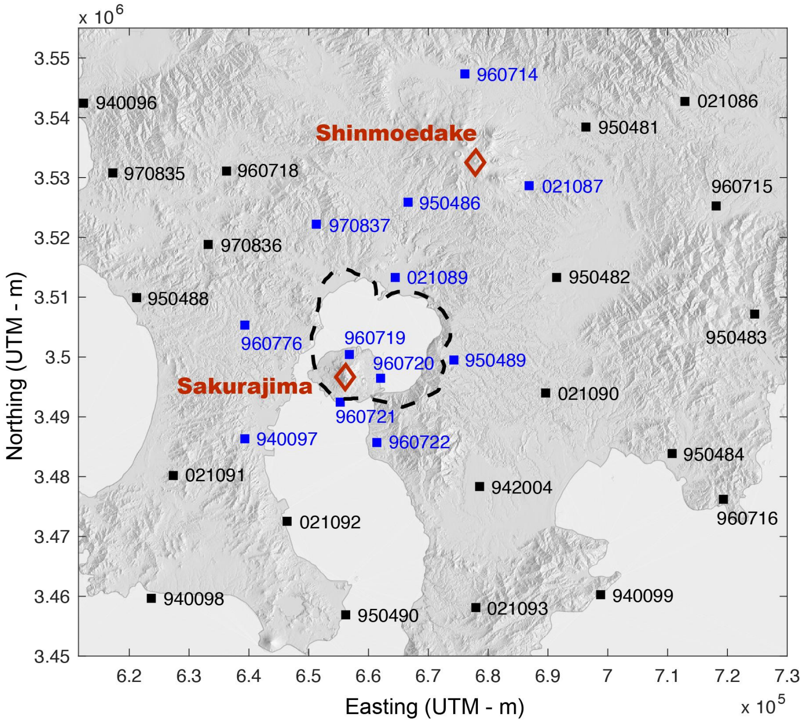

Fig. A1: Location of GNSS stations used in that study (GEONET Japanese network). 12 stations in blue were used to assess baseline changes and displacements in the volcanic areas. 20 stations in black, further from the 2 volcanoes were used to estimate tectonic displacements and common mode errors. Red diamonds indicate the location of Shinmoedake and Sakurajima volcanoes. The dashed-line indicates Aira caldera. Shaded relief map generated from a 10-m resolution Digital Elevation Model - Source: Geospatial Information Authority of Japan website (https://na01.safelinks.protection.outlook.com/?url=https%3A%2F%2Ffgd.gsi.go.jp%2Fdownload%2Fmenu.php&data=02%7C01%7Cebrothelande%40rsmas.miami.edu%7C2b7dc5ed125b4bed0bd108d5a5baa06f%7C2a144b72f23942d48c0e6f0f17c48e33%7C0%7C0%7C636597143364499667&sdata=f%2FDi1DA1G42vTxlTc2vgXYFMbAM5x%2B7POynk7mr1PEM%3D&reserved=0).

Appendix B of the supplementary material: Deformation models

Source	X - UTM (m)	Y - UTM (m)	Z (m)
Aira	660069	3501882	-11000
Sakurajima	657222	3495641	-4000
Kirishima	672984	3534914	-8570

Table B.1: Sources locations used in the model (from previous geodetic studies[1,2])

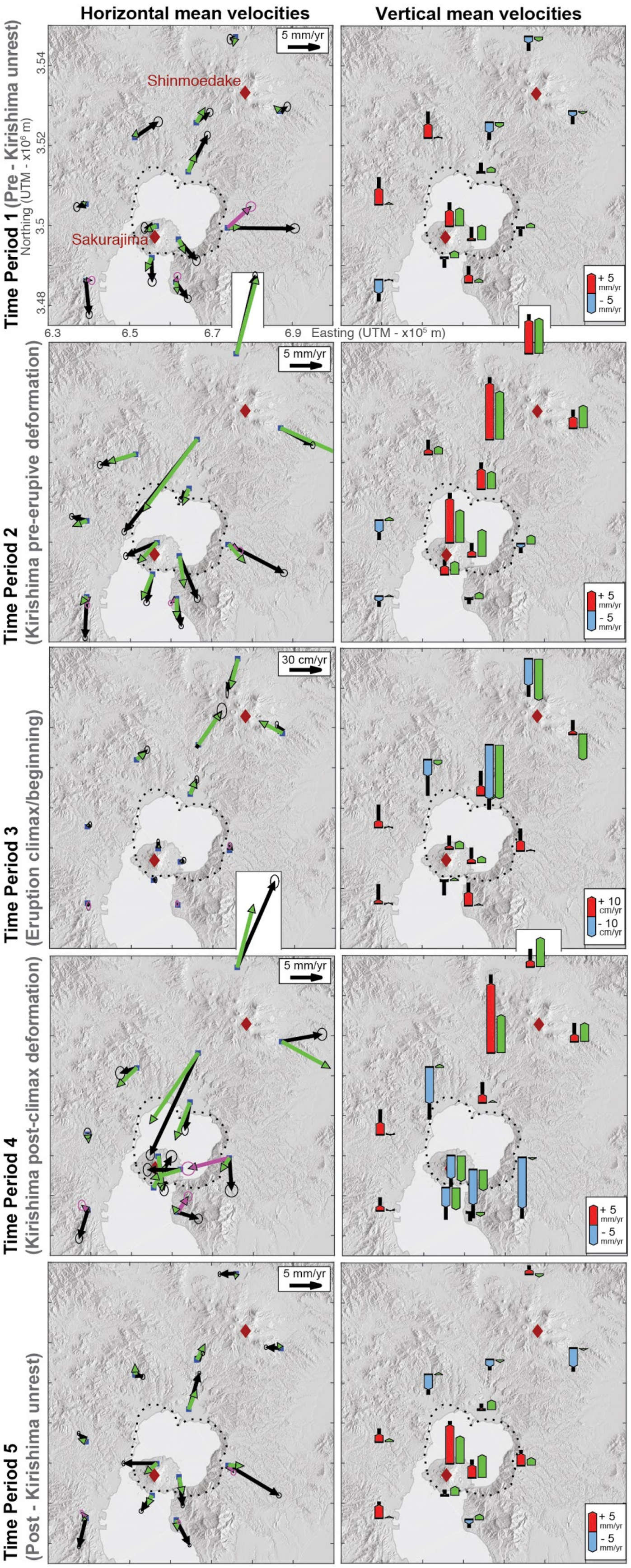

Horizontal mean velocities
Vertical mean velocities
Time Period 1 (Pre - Kirishima unrest)
Time Period 2 (Kirishima pre-erupive deformation)
Time Period 3 (Eruption climax/beginning)
Time Period 4 (Kirishima post-climax deformation)
Time Period 5 (Post - Kirishima unrest)
Shinmoedake
Sakurajima
Northing (UTM - x10^6 m)
Easting (UTM - x10^5 m)
3.54
3.52
3.5
3.48
6.3
6.5
6.7
6.9
5 mm/yr
30 cm/yr
+ 5 mm/yr
- 5 mm/yr
+ 10 cm/yr
- 10 cm/yr

Fig. B1 (above): Best-fit model velocities (green) plotted over observed velocities (black arrows and vertical bars) in Aira and Kirishima areas. Pink arrows represent re-estimated horizontal velocities at three stations after an additional correction (see Methods). Ellipses and black vertical bars represent 1-sigma uncertainties for horizontal and vertical velocities, respectively. Black dotted line: Aira caldera rim. Shaded relief map generated from a 10-m resolution Digital Elevation Model - Source: Geospatial Information Authority of Japan website (https://na01.safelinks.protection.outlook.com/?url=https%3A%2F%2Ffgd.gsi.go.jp%2Fdownload%2Fmenu.php&data=02%7C01%7Cebrothelande%40rsmas.miami.edu%7C2b7dc5ed125b4bed0bd108d5a5baa06f%7C2a144b72f23942d48c0e6f0f17c48e33%7C0%7C0%7C636597143364499667&sdata=f%2FDi1DA1G42vTxlTc2vgXYFMbAM5x%2B7POynk7mr1PEM%3D&reserved=0).

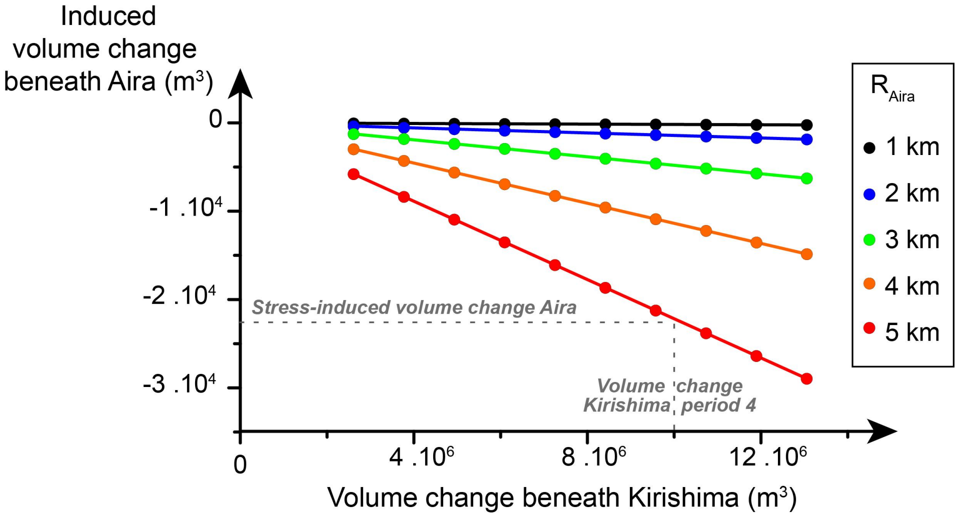

Fig. B2: Volume changes at Aira source induced by an expansion of Kirishima source. Different radii are considered for Aira, from 1 to 5 km. All values are very small in comparison to estimated volume changes at Aira during Kirishima post-climax inflation (approx. -1 .10^6 m^3)

References:

1. Nakao, S., Y., et al. Volume change of the magma reservoir relating to the 2011 Kirishima Shinmoe-dake eruption-Charging, discharging and recharging process inferred from GPS measurements, *Earth, planets and space* **65**(6), 505–515 (2013).
2. Iguchi, M. (2013). Magma Movement from the Deep to Shallow Sakurajima Volcano as Revealed by Geophysical Observations (Sakurajima Special Issue). *Bulletin of the Volcanological Society of Japan* **58**(1), 1-18.
